# The usefulness of narrow-band Imaging (NBI) in nasopharyngeal lesions—Validation of the Ni NBI classification dedicated for vascular pattern in the nasopharynx

**DOI:** 10.1371/journal.pone.0302043

**Published:** 2024-06-17

**Authors:** Hanna Klimza, Bartosz Maćkowiak, Kacper Zagaja, Joanna Jackowska, Jacek Banaszewski, Małgorzata Wierzbicka

**Affiliations:** 1 Regional Specialist Hospital Wroclaw, Research & Development Centre, Wrocław, Poland; 2 Department of Otolaryngology, Poznan University of Medical Sciences, Poznan, Poland; 3 Institute of Human Genetics, Polish Academy of Sciences, Poznań, Poland; 4 Wroclaw University of Science and Technology, Wroclaw, Poland; University of California, Davis, UNITED STATES

## Abstract

**Background:**

This study aims to explore the applicability of narrow-band imaging (NBI) involving the Ni classification for the diagnosis of nasopharyngeal mucosal lesions in order to distinguish malignant tumours (NPT) from non-malignant lesions.

**Methods:**

Each patient (n = 53) with a suspected nasopharyngeal lesion underwent a trans-nasal flexible video endoscopy with an optical filter for NBI. We assessed the suspected area using white light imaging (WLI) in terms of location and morphology as well as the vascular pattern (using Ni classification of nasopharyngeal microvessels) and surrounding tissue by using NBI. Based on the results of the NBI and WLI, patients were classified into "positive" or "negative" groups. All lesions of the nasopharynx were biopsied and submitted for final histological evaluation.

**Results:**

NBI showed higher sensitivity, specificity, and accuracy than WLI. There was a significant correlation between the final histological result and the NBI pattern of the NPT: Chi^2^(1) = 31.34; p = 0.000001 and the WLI assessment of the NPT: Chi^2^(1) = 14.78; p = 0.00012.

**Conclusions:**

The assessment of the NPT in NBI using Ni NBI classification proved valuable in suspected mucosa assessment. NBI not only confirms the suspicious areas in WLI, but it also shows microlesions beyond the scope of WLI and allows for proper sampling.

## Introduction

Narrow band imaging (NBI) is an advanced diagnostic tool which enhances the contrast between the microvascular pattern and the surrounding tissue using bandwidth filters in a red-green-blue sequential illumination system: blue light (wavelength peak **λ** of 400 to 430 nm) and green light (wavelength peak **λ** of 515 to 555 nm), which correspond to the absorption peaks of haemoglobin [1]. The main indicators of the pathological mucosa in NBI are intraepithelial papillary capillary loops (IPCs). In recent years, many authors have demonstrated the effectiveness of NBI in the detection of tumours in the gastrointestinal tract [2, 3], early glottic cancers [4–6], as well as in assessing and stratifying cancer risk in laryngeal leukoplakia [1, 7, 8] by means of appropriately validated classifications [9, 10]. Evaluating the vascular pattern in NBI requires a pertinent long learning curve and classifications dedicated to a specific head and neck region [11].

In the 2017 NBI classification, Ni et al. proposed a new classification of the nasopharyngeal mucosal NBI microvessel pattern in the differential diagnosis between benign and malignant nasopharyngeal tumours (NPT) [12]. The gold standard in confirming nasopharyngeal carcinoma is the histological result from a biopsy guided endoscopically using WLI. The large and exophytic nasopharynx tumours are not problematic in terms of the clinical diagnosis, unlike small and superficial ones; thus, it is vital to continuously improve the diagnostic tools, including the use and validation of NBI.

In view of the aforementioned findings, we decided to assess the utility of NBI in nasopharynx tumours using the new classification [12] by comparing the results from WLI, NBI, and the final histology results. In another measurement, it was assessed whether NBI could improve the diagnostics in early nasopharyngeal carcinoma.

## Material and methods

The presented study involved 53 patients with suspected NPT detected by MRI, CT, or nasopharyngeal endoscopy between April 2017 and December 2022 at the Department of Otolaryngology at Poznan University of Medical Sciences, a tertiary referral centre. The demographic and clinical characteristics of the subjects are presented in Table 1. Inclusion criteria comprised: patient with NPT tumour detected by MRI, CT, or nasopharyngeal endoscopy. The patients with previous head and neck malignancies and a history of head and neck irradiation were excluded from the study due to the abnormal microvessel pattern, which was unsuitable for NBI.

**Table 1:** Simplified diagnostic criteria used during white light imaging and narrow band imaging assessment by ENT specialists–classification of lesions.

Diagnostic criteria			
White light imaging (WLI)		Narrow-band imaging (NBI)	
Positive (WLI+)	Negative (WLI-)	Positive (NBI+)	Negative (NBI-)
nodule	adenoid	Type V acc. to Ni classification	Type I, II, III, IV acc. to Ni classification
cauliflower	cyst		
ulceration	reddish, smooth mucosa		
protrusion			

Each patient with a suspected NPT underwent a trans-nasal flexible video endoscope (Olympus Medical System Corporation, Tokyo, Japan) with an optical filter for NBI. Initially, we assessed the tumour using WLI in terms of location (posterior nostril, superior and posterior wall, Rosenmüllers’ recess, torus tuberous) and morphology. Subsequently, the vascular pattern of the nasopharyngeal tumour and surrounding tissue was evaluated by means of NBI. For the assessment of the vessels in NBI, the Ni classification of nasopharyngeal microvessels was applied [Ni 2017]. The vascular patterns of the nasopharyngeal mucosa were classified into five types on the basis of the intraepithelial papillary capillary loops (IPCLs) morphology [12].

All patients were classified into "positive" or "negative" groups according to the WLI and NBI results, as assessed by two ENT specialists (J.J and H.K). The diagnostic criteria defining the NBI(+) lesion characteristics were as follows: IPCLs were dilated, elongated, and distorted, and presented as distinctly irregular in appearance matching type V according to the 2017 Ni NBI classification.

The following diagnostic criteria were adopted defining the WLI(+) lesion characteristics on endoscopic examination: nodules, cauliflower, ulcers, and tumour protrusion. In contrast, the characteristics of WLI(-) lesions included: adenoid, cyst, light reddish, and smooth mucosa. The diagnostic criteria defining the “positive” and “negative” characteristics of the lesions according to WLI and NBI are presented in Table 1.

All lesions, detected by WLI or NBI endoscopy, were biopsied under local anesthesia (10% lidocaine spray). The specimens were preserved in 10% formalin and were submitted for final histological evaluation.

### Ethical approval

All procedures performed in studies involving human participants were in accordance with the ethical standards of the institutional and national research committee, as well as with the 1964 Helsinki Declaration and its later amendments or comparable ethical standards. The Bioethics Committee of Poznan University of Medical Sciences approved the study protocol (approval number 472/15).

### Informed consent

Informed written consent was obtained from all participants in the study.

## Results

Table 2 presents the layout of patients in the course of the study, where patients were assigned to Ni NBI classification type.

**Table 2:** Patient distribution during the study, according to the classification described in Table 1.

	WLI		NBI	
lesion	WLI **+**	WLI **-**	NBI **+**	NBI **-**
total	19 (35.85%)	34 (64.15%)	23 (43.40%)	30 (56.60%)
benign	4 (12.90%)	27 (87.10%)	3 (9.68%)	28 (90.32%)
malignant	15 (68.18%)	7 (32.82%)	20 (90.91%)	2 (9.09%)
	*p<0*.*00012*	*Chi*^*2*^ *= 14*.*78*	*p<0*.*000001*	*Chi*^*2*^ *= 31*.*34*

Among 53 patients who presented nasopharyngeal lesions, 19/53 (35.85%) showed a WLI(+) lesion characteristics and 34/53 (64.15%) demonstrated a WLI(-) characteristic of the lesion. Furthermore, in terms of NBI, 23/53 (43.40%) subjects were assessed as NBI(+) and corresponded to type V according to the Ni NBI classification. In contrast, NBI(-) pattern was observed in 30/53 (56.60%) participants, in which group 12/30 (22.64%) were classified as type I; 14/30 (26.41%) matched type II; 2/30 (3.77%) corresponded to type III, and 4/30 (7.55%) to type IV according to the Ni classification.

In all patients, the lesions assessed using WLI and NBI were compared with the final histological evaluation. In the group of 30 patients with NBI(-) vascular patterns 28 (90.32%) lesions were histologically classified as benign, however, in 2 (9.09%) cases the lesions were found to be malignant. Out of 23 patients with NBI(+) vascular patterns, in 20 (90.91%) patients malignant lesions were diagnosed and in 3 (9.68%) subjects the lesions were benign. There was a significant correlation between the NBI pattern of the nasopharyngeal lesions and the final histological results: Chi^2^(1) = 31.34; p = 0.000001.

Among 34 patients presenting a WLI(-) nasopharyngeal evaluation, in 27/34 (87.10%) patients histologically benign lesions were observed, and 7/34 (31.82%) subjects demonstrated malignant lesions. Out of 19 patients with a WLI(+) evaluation of the nasopharynx, the lesions were determined as benign in 4/19 (12.90%) patients. However, in 15/19 (68.18%) cases malignant lesions were found. There was a significant correlation between the WLI assessment of the nasopharyngeal lesion and the final histological result: Chi^2^(1) = 14.78; p = 0.00012.

The presented analysis (Table 3) showed that WL sensitivity, specificity, and accuracy were 87.10%, 68.18%, and 79.25%, respectively, whereas in terms of NBI it was 90.91%, 90.32%, and 90.57%, respectively. There was no statistically significant difference in age (p = 0.320805), gender (p = 1.32877), and lesion localization (p = 3.51642).

**Table 3:** Statistical analysis of NBI and WLI assessment.

Method	Sensitivity	Specificity	Disease prevalence	Positive Predictive Value	Negative Predictive Value	Accuracy
NBI	90.91%	90.32%	41.51%	86.96%	93.33%	90.57%
WLI	87.10%	68.18%	58.49%	79.41%	78.95%	79.25%

## Discussion

The objective of our research is to improve the diagnosis of early nasopharyngeal cancer (NPC), an insidious location, referred to as the blind spot, as well as to improve the differentiation of malignant and benign mucosal lesions. Moreover, our goal is also to identify the optimal point for obtaining samples from this technically difficult and demanding anatomical location.

NPC constitutes an epithelial carcinoma, although distinctly different from other epithelial head and neck tumours. It is a relatively uncommon tumour, estimated at 2.1 to 0.4 cases per 100 000 in Asia and Europe. NPC is endemic cancer, since more than 70% of new cases are found in Asia, predominantly in men. The main location of this cancer is the pharyngeal recess, i.e. Rosenmüllers’ fossa [13, 14], which was also observed in our study—approximately 90% of NPC were localized in this recess. Thus, this site requires particular emphasis on the refined evaluation of the mucosa in the screenings conducted in the high-risk population, or for second primary tumours [15]. The key issue is that the conventional WLI does not detect subtle changes in morphology, or in the mucosal blood vessels in the nasopharyngeal region. Furthermore, it is impossible to differentiate between nasopharyngeal carcinoma and chronic nasopharyngeal mucosal inflammation based on the colour and morphologic (apophysis or anabrosis) abnormalities using WLI [16]. This, in turn, may result in the misdiagnosis, or a failure to identify nasopharyngeal carcinoma.

The primary objective of our study was to compare the results between our findings and the prior studies which explored the effectiveness of using NBI. The strength of our study was to prove the applicability of NBI and its superiority over conventional WLI endoscopy in the precise diagnosis of NPT, which is consistent with the earlier reports. One of the studies described the diagnostic performance of WLI, NBI endoscopy, and endoscopic iodine staining for NPT. Interestingly, it showed significantly improved sensitivity, specificity, and positive predictive value compared to any procedure alone; in particular, NBI endoscopy and endoscopic iodine staining alone or combined demonstrated great clinical relevance [17].

The introduction of the Ni classification for nasopharyngeal NBI allows for organizing the "pathologies" visible in this area [12]. The superficial microvessel patterns of the nasopharyngeal mucosa were classified into five types based on the morphology of the intraepithelial papillary capillary loop (IPCL) identified by NBI, ranging from mild Type I to Types IV and V—the most suspected of malignancy. Type I was characterized by the invisible IPCLs, microvascular networks containing oblique and arborescent vessels, while Type V showed dilated, elongated, and distorted IPCLs, clearly irregular, twisted, brownish line shapes with snake-, earthworm-, or branch-like shapes [12]. Previous studies based on this classification suggest that the sensitivity and specificity of NBI endoscopy for nasopharyngeal lesions are higher than the conventional WLI [18]. According to the research conducted by Ni et al., the characteristics of NPC under NBI endoscopy mainly appeared as type V. The sensitivity and specificity of type V in the diagnosis of NPC were 79.5%, and 91.3%, respectively, whereas the diagnostic sensitivity and specificity of WLI endoscopy for NPC were 85.2% and 51.3% [12]. The diagnostic specificity of NBI was significantly higher than those of WLI.

Our results were similar to those cited above and showed that white light sensitivity, specificity, and accuracy were 87.10%, 68.18%, and 79.25%, respectively, whereas narrow-band imaging was 90.91%, 90.32%, and 90.57%, respectively. These findings suggested that NBI endoscopy demonstrated a higher specificity and sensitivity in diagnosing NPC.

Yet another issue is that the diagnostic accuracy of NBI has been found to be similar in different upper airway and upper digestive tract locations, assessment types, endoscope systems, as well as in the use of high-definition visualization [19, 20]. Thus, NBI may represent a vital additional tool for differentiating between benign and malignant lesions in the nasopharynx [19]. The recommendations for the diagnosis, treatment, and follow-up of NPT in the regions where NPC is endemic, include the use of plasma EBV DNA with a primer/probe assay targeting the BamHI-W region of the EBV genome, performed in duplicate and coupled with endoscopic examination and MRI. This study demonstrated a sensitivity and specificity in screening NPC of 97.1% and 98.6%, respectively [20]. The recommended endoscopic methods in the abovementioned areas include WLI and NBI [21].

Moreover, empirical findings emphasise that incorporating NBI, as a promising tool to differentiate non-malignant from malignant nasopharyngeal lesions, into routine endoscopic procedures in groups with higher susceptibility to NPC, contributes to a considerable reduction in the incidence of superfluous biopsies. It is of note that the observed outcome has the potential to significantly accelerate the diagnostic path, particularly in cohorts predisposed to NPC [22]. In addition, NBI can also constitute a relevant adjunct to constructed deep convolutional neural network-based artificial intelligence (AI) systems for detecting NPC with the use of archived nasopharyngoscopic images [23].

The strength of our study was also demonstrating the applicability of NBI and its superiority over conventional WLI endoscopy in the precise diagnosis of NPT. Nevertheless, the number of patients in the study group was limited. NBI may facilitate early detection or diagnosis of NPC, yet the authors were unable to substantiate this in data, since the limitation of our study was that it needed to include a healthy cohort in order to prove the screening power of this tool.

## Conclusions

The assessment of the nasopharyngeal lesions in NBI by means of Ni classification was found to be a valuable tool in the assessment and evaluation of suspicious areas of the mucosa. NBI confirmed the suspicious areas in white light and showed microlesions beyond the scope of white light. Given the promising results, further research including a wider population of patients is necessary.

## References

[1] Staníková L, Šatanková J, Kučová H, Walderová R, Zeleník K, Komínek P. The role of narrow-band imaging (NBI) endoscopy in optical biopsy of vocal cord leukoplakia. Eur Arch Otorhinolaryngol. 2017 Jan;274(1):355–359. doi: 10.1007/s00405-016-4244-6 Epub 2016 Aug 11. . 27515705

[2] Iwamuro M, Okuda M, Yumoto E, Suzuki S, Shirakawa A, Takata K, et al. Magnifying endoscopy for intestinal follicular lymphoma is helpful for prompt diagnosis. Gut Liver. 2013 Mar;7(2):258–61. doi: 10.5009/gnl.2013.7.2.258 Epub 2013 Mar 14. ; PMCID: PMC3607784. 23560166 PMC3607784

[3] Nonaka K, Ishikawa K, Arai S, Nakao M, Shimizu M, Sakurai T, et al. A case of gastric mucosa-associated lymphoid tissue lymphoma in which magnified endoscopy with narrow band imaging was useful in the diagnosis. World J Gastrointest Endosc. 2012 Apr 16;4(4):151–6. doi: 10.4253/wjge.v4.i4.151 ; PMCID: PMC3329616. 22523617 PMC3329616

[4] Piazza C, Cocco D, De Benedetto L, Bon FD, Nicolai P, Peretti G. Role of narrow-band imaging and high-definition television in the surveillance of head and neck squamous cell cancer after chemo- and/or radiotherapy. Eur Arch Otorhinolaryngol. 2010 Sep;267(9):1423–8. doi: 10.1007/s00405-010-1236-9 Epub 2010 Mar 30. . 20352239

[5] Watanabe A, Taniguchi M, Tsujie H, Hosokawa M, Fujita M, Sasaki S. The value of narrow band imaging for early detection of laryngeal cancer. Eur Arch Otorhinolaryngol. 2009 Jul;266(7):1017–23. doi: 10.1007/s00405-008-0835-1 Epub 2008 Nov 4. . 18982341

[6] Irjala H, Matar N, Remacle M, Georges L. Pharyngo-laryngeal examination with the narrow band imaging technology: early experience. Eur Arch Otorhinolaryngol. 2011 Jun;268(6):801–6. doi: 10.1007/s00405-011-1516-z Epub 2011 Feb 15. . 21327999

[7] Pietruszewska W, Morawska J, Rosiak O, Leduchowska A, Klimza H, Wierzbicka M. Vocal Fold Leukoplakia: Which of the Classifications of White Light and Narrow Band Imaging Most Accurately Predicts Laryngeal Cancer Transformation? Proposition for a Diagnostic Algorithm. Cancers (Basel). 2021 Jun 30;13(13):3273. doi: 10.3390/cancers13133273 ; PMCID: PMC8268866. 34208811 PMC8268866

[8] Klimza H, Jackowska J, Tokarski M, Piersiala K, Wierzbicka M. Narrow-band imaging (NBI) for improving the assessment of vocal fold leukoplakia and overcoming the umbrella effect. PLoS One. 2017 Jun 29;12(6):e0180590. doi: 10.1371/journal.pone.0180590 ; PMCID: PMC5491250. 28662209 PMC5491250

[9] Ni XG, Zhu JQ, Zhang QQ, Zhang BG, Wang GQ. Diagnosis of vocal cord leukoplakia: The role of a novel narrow band imaging endoscopic classification. Laryngoscope. 2019 Feb;129(2):429–434. doi: 10.1002/lary.27346 Epub 2018 Sep 19. . 30229933

[10] Arens C, Piazza C, Andrea M, Dikkers FG, Tjon Pian Gi RE, Voigt-Zimmermann S, et al. Proposal for a descriptive guideline of vascular changes in lesions of the vocal folds by the committee on endoscopic laryngeal imaging of the European Laryngological Society. Eur Arch Otorhinolaryngol. 2016 May;273(5):1207–14. doi: 10.1007/s00405-015-3851-y Epub 2015 Dec 17. . 26677852

[11] Yeung DC, Vlantis AC, Wong EW, Tong MC, Chan JY. A meta-analysis of narrow-band imaging for the diagnosis of primary nasopharyngeal carcinoma. F1000Res. 2018 Jun 18;7:759. doi: 10.12688/f1000research.15183.1 ; PMCID: PMC6039959. 30026934 PMC6039959

[12] Ni XG, Zhang QQ, Wang GQ. Classification of nasopharyngeal microvessels detected by narrow band imaging endoscopy and its role in the diagnosis of nasopharyngeal carcinoma. Acta Otolaryngol. 2017 May;137(5):546–553. doi: 10.1080/00016489.2016.1253869 Epub 2016 Nov 14. . 27841051

[13] Chen YP, Chan ATC, Le QT, Blanchard P, Sun Y, Ma J. Nasopharyngeal carcinoma. Lancet. 2019 Jul 6;394(10192):64–80. doi: 10.1016/S0140-6736(19)30956-0 Epub 2019 Jun 6. . 31178151

[14] Ferlay J, Ervik M, Lam F, et al. Global Cancer Observatory: Cancer Today. Lyon, France: International Agency for Research on Cancer; 2018. Available at: https://gco.iarc.fr/today. Accessed August 11, 2020.

[15] Filauro M, Paderno A, Perotti P, Marchi F, Garofolo S, Peretti G, et al. Role of narrow-band imaging in detection of head and neck unknown primary squamous cell carcinoma. Laryngoscope. 2018 Sep;128(9):2060–2066. doi: 10.1002/lary.27098 Epub 2018 Feb 2. . 29392723

[16] Si YF, Deng ZX, Weng JJ, Si JY, Lan GP, Zhang BJ, et al. A study on the value of narrow-band imaging (NBI) for the general investigation of a high-risk population of nasopharyngeal carcinoma (NPC). World J Surg Oncol. 2018 Jul 4;16(1):126. doi: 10.1186/s12957-018-1423-5 ; PMCID: PMC6032783. 29973209 PMC6032783

[17] Yang F, Huang N, Chen X, Wang M. Application of narrow band imaging and Lugol’s iodine staining in screening for nasopharyngeal carcinoma. World J Surg Oncol. 2023 Nov 30;21(1):376. doi: 10.1186/s12957-023-03258-5 ; PMCID: PMC10687887. 38037075 PMC10687887

[18] Madana J, Lim CM, Loh KS. Narrow band imaging of nasopharynx to identify specific features for possible detection of early nasopharyngeal carcinoma. Head Neck. 2015 Aug;37(8):1096–101. doi: 10.1002/hed.23705 Epub 2015 Jun 16. . 24710736

[19] Zhou H, Zhang J, Guo L, Nie J, Zhu C, Ma X. The value of narrow band imaging in diagnosis of head and neck cancer: a meta-analysis. Sci Rep. 2018 Jan 11;8(1):515. doi: 10.1038/s41598-017-19069-0 ; PMCID: PMC5765024. 29323235 PMC5765024

[20] Chung CS, Wu CY, Lin YH, Lo WC, Cheng PC, Hsu WL, et al. Screening and surveillance of esophageal cancer by magnifying endoscopy with narrow band imaging improves the survival of hypopharyngeal cancer patients. Front Oncol. 2024 Jan 23;13:1221616. doi: 10.3389/fonc.2023.1221616 ; PMCID: PMC10844580. 38322289 PMC10844580

[21] Bossi P, Chan AT, Licitra L, Trama A, Orlandi E, Hui EP, et al. Electronic address: clinicalguidelines@esmo.org; EURACAN. Nasopharyngeal carcinoma: ESMO-EURACAN Clinical Practice Guidelines for diagnosis, treatment and follow-up†. Ann Oncol. 2021 Apr;32(4):452–465. doi: 10.1016/j.annonc.2020.12.007 Epub 2020 Dec 25. . 33358989

[22] Wen YH, Zhu XL, Lei WB, Zeng YH, Sun YQ, Wen WP. Narrow-band imaging: a novel screening tool for early nasopharyngeal carcinoma. Arch Otolaryngol Head Neck Surg. 2012 Feb;138(2):183–8. doi: 10.1001/archoto.2011.1111 . 22351866

[23] Wang SX, Li Y, Zhu JQ, Wang ML, Zhang W, Tie CW, et al. The Detection of Nasopharyngeal Carcinomas Using a Neural Network Based on Nasopharyngoscopic Images. Laryngoscope. 2024 Jan;134(1):127–135. doi: 10.1002/lary.30781 Epub 2023 May 31. . 37254946

